# Making GFP count: a validated framework for absolute protein quantification in precision fermentation

**DOI:** 10.1007/s00253-026-13734-z

**Published:** 2026-02-04

**Authors:** Christina Peternell, Philipp Noll, Annette Brümmer-Rolf, Marius Henkel

**Affiliations:** Cellular Agriculture, TUM School of Life Sciences, Technical University of Munich, Gregor-Mendel-Str. 4, Freising, 85354 Germany

**Keywords:** Precision fermentation, Bioprocess optimization, High-throughput protein quantification, Green fluorescent protein, Absolute protein concentration method, Fluorescence signal standardization and validation

## Abstract

Precise quantification of recombinant proteins is essential for assessing and comparing expression efficiency and optimizing production processes. Fluorescent proteins have emerged as powerful tools for real-time monitoring of gene expression and protein tracking. However, standardized and validated methods for their quantification, particularly for the widely used green fluorescent protein, remain limited. To date, no universally adopted protocol has emerged. This study presents a high-throughput method for the quantification of recombinantly produced Emerald Green Fluorescent Protein (EmGFP) based on direct fluorescence measurements of the cell suspension while quantifying and integrating potential effects of signal attenuation. The workflow uses solely standard laboratory equipment, ensuring broad accessibility and easy implementation. Moreover, *in-house* EmGFP standard preparation and quantification is described. The method was validated according to FDA guidelines “Analytical Procedures and Methods Validation for Drugs and Biologics,” addressing the requirements of linearity, limit of detection (LOD), limit of quantification (LOQ), precision, accuracy, and recovery rate. Investigation was conducted using *Escherichia coli* BL21 cells expressing EmGFP, widely available sodium fluorescein as a chemical standard, commercial GFP, and an *in-house* EmGFP standard. A robust correlation (linear fitting, *R*^2^ 0.96) of the EmGFP concentration and relative fluorescence units (RFU) was established, enabling efficient and high-throughput fluorescence quantification using a standardized workflow in a microtiter-based format suitable for the application in comparative studies across different expression constructs, conditions, and scales. By enabling absolute quantification of fluorescent proteins, this method supports both real-time bioprocess optimization and broader applications in protein production research.

**Supplementary Information:**

The online version contains supplementary material available at 10.1007/s00253-026-13734-z.

## Introduction

Fluorescent proteins (FPs) are indispensable tools in life sciences and biotechnology. Since the discovery of green fluorescent proteins (GFP) in the jellyfish *Aequorea victoria*, they have enabled researchers to study gene expression, localization, and dynamics (Chalfie et al. 1994; Tsien 1998; Shimomura 2005; Day and Davidson 2009). Among the many available FPs, green fluorescent protein (GFP) and its engineered variants such as enhanced GFP (eGFP) or emerald GFP (EmGFP) are most widely used. Their stability, visibility, improved brightness, photophysical properties, and ease of genetic fusion to proteins of interest provide a broad application range (Inouye and Tsuji 1994; Zimmer 2002; Wachter 2006). GFPs have evolved from qualitative markers to quantitative reporters. Their main advantage lies in their intrinsic fluorescence (i.e., no exogenous substrate is needed), and the signal is directly proportional to the amount of protein within the linear detection range (Cehelnik et al. 1975; Cubitt et al. 1995; Day and Davidson 2009). Therefore, GFPs are widely used in synthetic biology, strain engineering, and high-throughput screening of genetic constructs (Chalfie et al. 1994; Zimmer 2002; Wachter 2006). However, limitations remain: although GFP signals can be measured simply and fast via fluorescence, the values are recorded in arbitrary or relative fluorescence units (AUs or RFUs), which vary between devices and equipment (Cehelnik et al. 1975; Scholz et al. 2000; Gaigalas et al. 2001; Beal et al. 2018). Furthermore, it is worth highlighting that fluorescence measurement is a very sensitive method, being influenced by various factors such as the correct folding of the protein itself, the presence of oxygen or interfering (cell) compounds, or measurements in intact cells causing quenching effects (Zimmer 2002; Jung, Kie-Chul et al. 2005; Wachter 2006; Drepper et al. 2010). Common approaches are describing fluorescent intensity in relation to cell populations, biomass concentrations, or used standards (Lissemore et al. 2000; Scholz et al. 2000; Csibra and Stan 2022). Methods such as calibration with fluorescein, serving as a chemical standard molecule for GFP and its mutants, can convert RFUs into molecules of equivalent fluorescein (MEFL), but do not account for absolute protein quantification (Fedorec et al. 2020). An alternative approach is reported by Csibra and Stan (2022), who used in vivo calibration curves and synthetic genetic programs to link reporter protein output directly to molecular concentrations, allowing modular and quantitative monitoring of expression levels across engineered cell populations. Their method highlights how system-wide calibration and standardization can support more predictive design in synthetic biology applications. Nevertheless, the lack of standardization remains a challenge and hinders reliable comparison and determination of absolute units (Gaigalas et al. 2001). To address this, a method that converts in-cell measurement of RFU into absolute protein concentrations is needed. The work presented here describes a method for high-throughput quantification of EmGFP expression in *Escherichia coli* BL21 in microtiter plate scale and widely accessible laboratory techniques. Relative fluorescence units along with host cell protein quantification and SDS-PAGE analysis are combined to establish a correlation between in-cell measurement of RFU and absolute protein concentration [mg/L]. Although the presented workflow requires a one-time validation for specific experimental conditions, it enables standardized fluorescence quantification across devices, expression levels, and cultivation formats ranging from microtiter plates and shake flasks to high-cell density batch and fed-batch bioreactor processes. Its unique characteristic is a standardized procedure for low sample volumes and suitability for subsequent high-throughput quantification. The method further facilitates rapid comparison of productivity between different strains and cultivation conditions, as well as the determination of process-relevant parameters such as specific production rates and product yields based on protein mass rather than arbitrary units. It also supports the calibration of soft sensors detecting real-time fluorescence measurements used for absolute FP quantification in (bioreactor) vessel cultivations (Jones et al. 2004). Jones et al. (2004) developed an on-line fluorescence sensor for monitoring GFP fluorescence as RFU in a 20-L bioreactor setup, using affinity chromatography and liquid chromatography-mass spectrometry (LC-MS) for off-line absolute protein quantification. In contrast, our workflow enables the correlation of RFU signals with absolute fluorescent protein concentrations using accessible laboratory techniques such as absorbance-based assays and SDS-PAGE.

Furthermore, its application extends to studies on novel expression systems and process optimization (Terpe 2006; Treinen et al. 2025), such as bulk proteins in precision fermentation. Moreover, the method holds significant relevance in the context of expressing complex or modified fluorescent proteins, where a standardized, accessible, and scalable workflow for quantitative data detection is lacking or limited.

By combining fluorescence, host cell protein analysis, and a calibration procedure based on different standards, a robust and reproducible method to convert RFU [-] into absolute EmGFP concentrations [mg/L] is provided. Through reliable absolute measurements, this approach supports both real-time monitoring during bioprocesses and broader applications in protein engineering and production research. The presented method is distinct in its combination of microtiter plate format and routine laboratory practices.

## Materials and methods

### Microorganism and plasmids

A genetically modified *E. coli* BL21 was used for evaluating the expression of EmGFP reporter under the translational control of a constitutive promoter J23108 from the Anderson promoter family (Anderson et al. 2010). Plasmids were transferred by heat shock into chemically competent *E. coli* DH5α (Thermo Scientific Inc., Waltham, MA, USA) for storage and amplification and chemically competent *E. coli* BL21 (formerly Stratagene CA, La Jolla, CA, USA) as the working and production strain. Transformants were stored at −80 °C in 20% (v/v) glycerol stocks.

### Plasmid construction

Plasmid construction was performed according to Noll et al. (2021). Plasmid map of *E. coli* BL21 pRSET_J23108_EmGFP is provided as Supplementary Information, Fig. S1. The promoter and the 5′ untranslated region (UTR) up to codon nine of the gene of interest (EmGFP, including a N-terminal his-tag for purification) were synthesized and obtained in a pEX-A128 plasmid from Eurofins Genomics Germany GmbH Hamburg, Germany. The segment of interest was amplified, digested (R0655S *PciI* and R0581S *BseRI*, New England Biolabs GmbH, Frankfurt am Main, Germany), and ligated (T4 DNA Ligase EL0014, Thermo Scientific Inc., Waltham, MA, USA) into the working plasmid pRSET-EmGFP (V35320, Invitrogen by Thermo Scientific, Waltham, MA, USA). The host cell *E. coli* BL21 without a plasmid was used as a control.

### Chemicals and substances

Chemicals used in this study were purchased from Carl Roth GmbH & Co. KG (Karlsruhe, Germany), VWR International GmbH (Darmstadt, Germany), Bio-Rad Laboratories, Inc. (Hercules, CA, USA), and Merck KGaA (Darmstadt, Germany) if not stated otherwise.

### Media preparation

For *E. coli* BL21 pRSET_J23108_EmGFP cultivation, ampicillin was added to all media to a final concentration of 0.1 g/L. The pH of all media was adjusted to pH 7.0 ± 0.2 with 1 M NaOH or 4 M H_3_PO_4_. Lysogeny broth (LB) medium (tryptone 10 g/L; yeast extract 5 g/L; NaCl 10 g/L; agar 15 g/L) was prepared according to Bertani (1951). M9 media was prepared according to Henkel et al. (2015). Phosphate-buffered saline (PBS) (NaCl 8.0 g/L; KCl 0.2 g/L; Na_2_HPO_4_ 1.42 g/L; KH_2_PO_4_ 0.27 g/L; pH adjusted to 7.4 ± 0.2) was prepared according to Dulbecco and Vogt (1954).

### Overview of experimental workflow

**Fig. 1:**
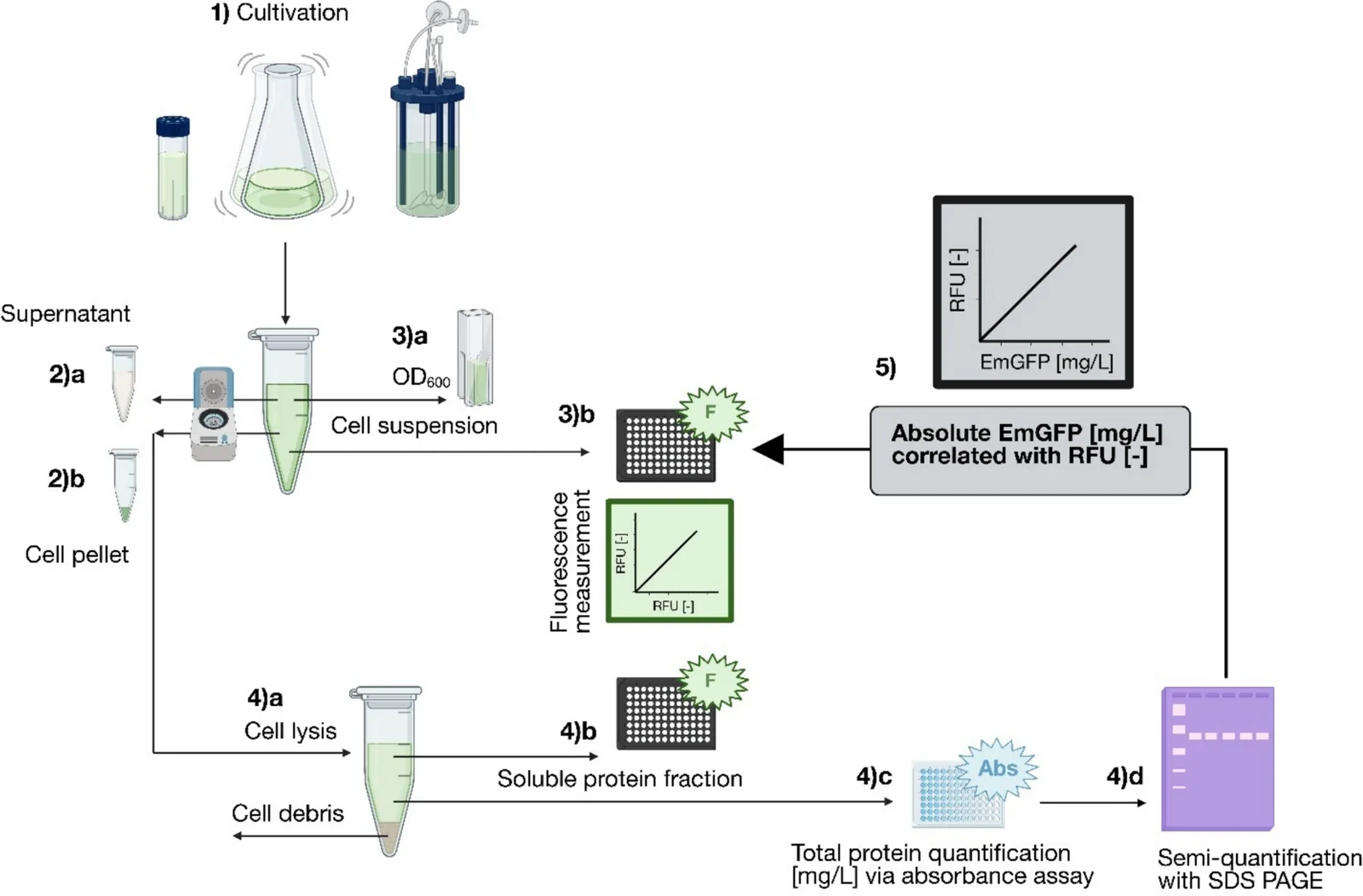
Experimental walkthrough for establishing a correlation of relative fluorescence units (RFU) [-] with EmGFP [mg/L] concentration. (1) bacterial cultivation, here in shake flasks. Sampling and cell suspension analysis after centrifugation are conducted as follows: (2a) supernatant for substrate quantification, (2b) cell pellet for cell lysis separating into cell debris and soluble protein fraction. Cell suspension is used for (3a) determination of biomass concentration via OD_600_ measurement and (3b) fluorescence measurement determining EmGFP expressed in vivo (in-cell measurement). Soluble protein fraction is analyzed as follows: (4a) cell lysis step, (4b) fluorescence measurement determining EmGFP in soluble protein fraction, (4c) determination of total protein via absorbance assays, (4d) analysis of EmGFP with SDS-PAGE. (5) Correlation establishment of RFU [-] to absolute EmGFP concentration [mg/L]. Created in BioRender. Peternell, C. (2026) https://BioRender.com/xujlk8x

An overview of the practical workflow for the quantification of EmGFP concentration is shown in Fig. 1. This multi-layered procedure ensures robustness and comparability across instruments and applications. This correlation approach is particularly suitable for subsequent high-throughput screening of genetically modified strains or constructs in bacterial systems expressing GFP-like or other fluorescent proteins, as it enables the evaluation and classification of expression levels.

### Cultivation in shake flasks

All shake flask cultivations were carried out in a shake incubator (Minitron, Infors AG, Bottmingen, Switzerland) with a shaking diameter of 25 mm and at 160 rpm (Fig. 1 (1)). Twenty-five milliliters of LB media in a 250-mL baffled shake flask with three side baffles was inoculated with 25 μL glycerol stock of *E. coli* BL21 pRSET_J23108_EmGFP or *E. coli* BL21, and a sterile media control was incubated for 12 h at 37 °C and 160 rpm. One hundred milliliters of M9 media in a 1-L baffled shake flask with three side baffles was inoculated to an optical density (OD_600_) of 0.1 from the pre-culture in LB media. The main culture in M9 media and a sterile media control were incubated at 35 °C for 24 h. Cultivations of the main cultures were carried out in triplicates.

### Sample collection and processing

Samples (2 mL) were drawn every 2 h if not stated otherwise over 24 h. Cell turbidity was determined (Fig. 1 (3a)) via optical density measurement at a wavelength of 600 nm in a spectrophotometer (Ultrospec™ 10 Cell Density Meter, Harvard Bioscience Inc., Holliston, MA, USA). The correlation factor for biomass concentration and OD_600_ was determined with 0.334 ± 0.003 according to Geissler et al. (2019) with the following adaptations: three times 40 mL of culture harvested from the exponential growth phase were filled in pre-dried (110 °C for 24 h, ST 6120, Heraeus Group, Hanau, Germany and 24 h cooled down in a desiccator) and pre-weighted (MC1 RC250 s, Satorius AG, Göttingen, Germany) 50-mL centrifugation tubes (XX99.1, Carl Roth GmbH & Co. KG, Karlsruhe, Germany) and centrifuged for 10 min at 6000 rpm and 4 °C (75002555, Thermo Scientific Inc., Waltham, MA, USA). The supernatant was discarded, and the cell pellet was washed with 40 mL 0.9% (w/v) NaCl and centrifuged using the same conditions mentioned above. Washing was performed until the supernatant appeared transparent. Cell pellets, without supernatant, were dried at 110 °C for 24 h, cooled down in a desiccator for 24 h, and weighed in order to determine the conversion factor (OD_600_/biomass concentration).

Relative fluorescence units (RFU) [-] of well-mixed samples were determined using 200 μL sample volume per well (Fig. 1 (3b and 4b)). One milliliter of the sample was centrifuged at 16,000 × g for 6 min at 4 °C (Rotina 380R, Hettich GmbH & Co.KG, Tuttlingen, Germany), stored at −20 °C until continuing with substrate quantification (Fig. 1 (2a)) and the cell lysis step (Fig. 1 (2b)).

### Glucose quantification assay

To determine the glucose concentration, cell-free supernatants were analyzed (Fig. 1 (2a)) using the K_GLUC D-Glucose Assay Kit (GOPOD Format, 700004297, Megazyme Ltd., Wicklow, Ireland). Monitoring glucose levels throughout cultivation is essential for assessing substrate consumption and depletion. Furthermore, identifying the end of the exponential growth phase, i.e., the phase of recombinant protein production, helps to define the applicable range for the subsequently established in-cell measurement of RFU-absolute protein concentration correlation (see also Fig. 5). A microplate reader (Tecan Spark reader, Tecan Trading AG, Männedorf, Switzerland) with SparkControl Magellan and MethodEditor software (Tecan Group Ltd., Männedorf, Switzerland) was utilized to measure absorbance at a wavelength of 510 nm, with a settle time of 50 ms and 10 flashes. The assay was performed according to the manufacturers’ protocol in a 96-well plate (655.101, Greiner Bio-One GmbH, Frickenhausen, Germany), utilizing 1/12.5 of the volumes described in the manual. For determination, a glucose standard curve was generated using a dilution series of 0, 200, 400, 600, 800, and 1000 mg/L and corresponding absorption values (*R*^2^ > 0.99) provided in the Supplementary Information, Fig. S5. All samples were analyzed in technical duplicates.

### *In-house* EmGFP standard preparation

Cultivation of *E. coli* BL21 pRSET_J23108_EmGFP was performed in LB and M9 media for 24 h. The cell suspension was harvested and centrifuged for 30 min, 16,000 g, and 4 °C in an ultracentrifuge (75002555, Thermo Scientific Inc., Waltham, MA, USA). The lysis step was performed by resuspending the cell pellet with 375 μL lysis buffer (350 μL STET buffer: 10 mM Tris/HCl pH 8.0; 0.1 M NaCl; 1 mM EDTA; 5% (v/v) Triton X-100 and 25 μL lysozyme 10 mg/mL in 10 mM Tris/HCl pH 8.0 ± 0.1 solution) per milliliter cell suspension. Cell lysis was performed at 37 °C for 30 min in a thermo shaker (Hettich Lab Technology, Tuttlingen, Germany), a subsequent heat inactivation step at 80 °C for 1 min and followed by centrifugation at 4 °C, 30 min, and 16,000 g (Rotina 380R, Hettich GmbH & Co.KG, Tuttlingen, Germany). The supernatant containing host cell protein and EmGFP was sterile filtered with a PES filter (0.45 μm, 83.1826, Sarstedt AG und Co. KG, Nümbrecht, Germany) and used for protein separation and affinity based (6× his-tag linked to the EmGFP) purification with ÄKTA pure 25 (ÄKTA, Cytiva Life Sciences, Marlborough, MA, USA). Ten milliliters was applied on a 5-mL HisTrap column (Cytiva Life Sciences, Marlborough, MA, USA) using phosphate buffer (20 mM Na_3_PO_4_ pH 7.4 ± 0.2; 0.5 M NaCl; 5 mM imidazole) and collected in 5-mL fractions. After washing with phosphate buffer supplemented with 5 mM imidazole to remove proteins with nonspecific binding to the column, the EmGFP was eluted in a first step using phosphate buffer supplemented with 0.4 M imidazole followed by a second elution step with 0.5 M imidazole (a representative chromatogram is provided in the Supplementary Information, Fig. S13). For buffer exchange, a desalting column (Superdex200 10/300 GL, Marlborough, MA, USA) was used to exchange the elution buffer with a phosphate buffer without imidazole. Elution volume between 15.9 and 18.2 mL was collected as fraction “EmGFP standard” based on absorbance spectra at 280 nm and 487 nm and concentrated with Vivaspin 20, 10 kDa MWCO Polyethersulfone (Marlborough, MA, USA). The purified protein standard was quantified with several absorbance assays: at 595 nm (Bradford assay), absorbance at 280 nm and 487 nm (A_280_ and A_487_) using a photometer (NP80, Implen, Munich, Germany), absorbance at 562 nm (BCA assay), as well as with SDS-PAGE. Further characterization included monitoring of excitation and emission spectra as well as determination of relative fluorescence units (RFUs) [-] at different concentrations. To ensure consistency, all subsequent calculations were based on the concentration of 12.6 g/L determined via absorbance at 487 nm (A_487_).

*In-house* EmGFP standard was frozen in phosphate buffer at −20 °C in aliquots until further use.

### Fluorescence measurement

EmGFP monitoring was conducted as fluorescence measurement in a microplate reader (Tecan Spark reader, Tecan Trading AG, Männedorf, Switzerland) with SparkControl Magellan 3.0 and MethodEditor (Tecan Group Ltd., Männedorf, Switzerland). Relative fluorescence units (RFU) [-] of well-mixed samples were measured in a black 96-well plate (655.076, Greiner Bio-One GmbH, Frickenhausen, Germany) using 200 μL sample volume per well with excitation at 487 nm (fluorophore dropdown list: eGFP). Emission was recorded at 540 nm with a fixed gain set to 60 and a Z-position of 20,000 μm. For each time point, samples were measured as technical duplicates. In-cell measurement (cell suspension, Fig. 1 (3b)) and soluble protein solution (after cell lysis, Fig. 1 (4)b)) were measured respectively. As a (background) control, M9 media, PBS, 20% (v/v) glycerol, and cell lysis buffer (BugBuster Master Mix, 71456-3, Merck KGaA, Darmstadt, Germany) are measured. Additionally, a commercial GFP standard (14–392, Merck KGaA, Darmstadt, Germany, 1 g/L, purity =  > 70% after Ni-NTA-Agarose and SDS-PAGE), *in-house* EmGFP standard (12.6 g/L determination with A_487_, purity 78%, *n* = 4, after HisTrap column and SDS-PAGE, 3D structure provided in Supplementary Information, Fig. S12a), and 1 μM (equivalent to 0.376 mg/L) sodium fluorescein (Na-F*, CAS No. 518-47−8, Carl Roth GmbH & Co. KG, Karlsruhe, Germany, chemical structure provided in Supplementary Information, Fig. S12b) in 100 mM NaOH were measured for comparison. Samples that show signals above the value of 49,000 RFU (2 μM diluted 1:2 equivalent to 0.376 mg/L in 100 mM NaOH, see also Table 1) are diluted with PBS accordingly. A correlation is established of RFU_cellsuspension_ [-] and RFU_solubleproteins_ [-], visualizing differences of EmGFP fluorescence detection when measured in vivo and after cell lysis in vitro as released soluble protein solution. A correlation is established from the beginning of cultivation (here: t0, inoculation point) until the maximum RFUs are reached (here: t9, after 19 h of cultivation).

### Cell lysis

Cell lysis was performed by using all-in-one cell lysis buffer (BugBuster Master Mix, 71456-3, Merck KGaA, Darmstadt, Germany) containing a protein extraction reagent with a benzonase nuclease and a rLysozyme solution according to the manufacturer’s instructions. Additionally, a protease inhibitor reagent (Protease Inhibitor Cocktail Set III, EDTA-Free-Calbiochem, 539134, Merck KGaA, Darmstadt, Germany) was used. Cells were harvested from the fermentation broth by centrifugation at 16,000 × g for 6 min at 4 °C (Rotina 380R, Hettich GmbH & Co.KG, Tuttlingen, Germany), and the supernatant was removed and was stored at −20 °C. Subsequently, cell pellets from 1-mL centrifuged cell suspension were resuspended in 500 µL room temperature lysis buffer and 10 μL protease inhibitor reagent. Cell suspension was incubated in a thermo shaker (Hettich Lab Technology, Tuttlingen, Germany) at 200 U/min for 20 min at room temperature. Insoluble cell debris was separated by centrifugation at 16,000 × g for 20 min at 4 °C (Fig. 1 (4a)). Finally, the supernatant was transferred to a fresh tube and fluorescence measurement (see section “Fluorescence measurement,” Fig. 1 (4b)), protein determination (see section “Protein quantification,” Fig. 1 (4c)), and SDS-PAGE analysis (see section “Sodium dodecylsulphate polyacrylamide gel electrophoresis (SDS-PAGE),” Fig. 1 (4d)) were carried out. To confirm lysis efficiency, pellets of disrupted cells were resuspended in 1 mL PBS and qualitatively assessed with a microscope (Axiovert 135, Carl Zeiss Microscopy GmbH, Jena, Germany). One hundred microliters of the suspension was streaked out on LB agar plates (0.1 g/L Amp, pH 7.0 ± 0.2) and incubated at 37 °C over night for quantitative analysis.

### Protein quantification

Protein quantification (Fig. 1 (4c)) was performed using two commonly applied absorbance-based assays routinely used in laboratory practice for comparative analysis.

#### Bicinchoninic acid assay (BCA)

Protein quantification was performed with a bicinchoninic acid (BCA) kit in 96-well plates (71285, Merck KGaA, Darmstadt, Germany). A microplate reader (Tecan Spark reader, Tecan Trading AG, Männedorf, Switzerland) with SparkControl Magellan 3.0 software was utilized to measure absorbance at a wavelength of 562 nm, with a settle time of 50 ms and 10 flashes. For quantification, the absorbance of a bovine serum albumin (BSA) standard curve was recorded at 0, 25, 125, 250, 500, 750, and 1000 mg/L yielding an *R*^2^ > 0.99. Before absorbance measurement at 562 nm, standard and samples mixed with reagent were incubated in a thermo shaker (Hettich Lab Technology, Tuttlingen, Germany) at 37 °C, 200 U/min for 30 min. All samples were analyzed in technical duplicates.

#### Bradford assay

Protein quantification was performed with ROTI Quant assay according to Bradford (K015.1, Carl Roth GmbH & Co. KG, Karlsruhe, Germany). A microplate reader (Tecan Spark reader, Tecan Trading AG, Männedorf, Switzerland) with SparkControl Magellan 3.0 software was utilized to measure absorbance at a wavelength of 595 nm, with a settle time of 50 ms and 10 flashes. Protein content is determined by using the BSA standard curve (0, 20, 30, 40, 50, 60, 80, 100 mg/L) and corresponding absorption values (*R*^2^ > 0.99). The assay was performed according to the manufacturer’s protocol in a 96-well plate (655.101, Greiner Bio-One GmbH, Frickenhausen, Germany). Before absorbance measurement at 595 nm, the plate was incubated at room temperature for 5 min. All samples were analyzed in technical duplicates.

### Sodium dodecylsulphate polyacrylamide gel electrophoresis (SDS-PAGE)

To determine the amount of EmGFP in relation to the amount of host cell protein, a SDS-PAGE was carried out (Fig. 1 (4d)). Soluble protein solutions were mixed with reducing SDS sample buffers (1:1 or 2:1). Afterwards, samples were incubated in a thermo shaker (Hettich Lab Technology, Tuttlingen, Germany) at 95 °C, 200 U/min for 5 min and centrifuged at 10,000 g for 5 min. Precast gradient gels (Mini-PROTEAN TGX Precast Protein Gel 4–20% gel, 4568093, Bio-Rad Laboratories, Inc., Hercules, CA, USA) were loaded with 10 μL (1:1, 5 μL sample and 5 μL reducing buffer) or 15 μL (2:1, 10 μL sample and 5 μL reducing buffer) and placed in the gel chamber (Mini-PROTEAN Tetra Systems, Bio-Rad Laboratories, Inc., Hercules, CA, USA). The Precision Plus Protein Unstained Protein Standard (1610363, Bio-Rad Laboratories, Inc., Hercules, CA, USA) was used as a molecular weight marker (10–250 kDa). Additionally, purchased commercial GFP standard and *in-house* EmGFP standard (see section “Standards” in the “Materials and methods”; Table 1) were used as references. Gels were run at 150 V, 40 mA per gel, and 35 Watt by electrophoresis power supply (EPS 3501 XL, Amersham Pharmacia Biotech, Amersham, UK).

The gels were recorded using an image reader (Gel Doc XR+ Imager, Bio-Rad Laboratories, Inc., Hercules, CA, USA) at an exposure time of 4 s, and band intensities were quantified by densitometry using Image Lab software (version 6.1.0 build 7, 2020, Bio-Rad Laboratories, Inc., Hercules, CA, USA).

### Absolute EmGFP correlation establishment

RFUs_cellsuspension_ [-] in relation to absolute EmGFP concentrations [mg/L] are plotted as schematically illustrated in Fig. 1 (5). As previously described, a correlation is established from the beginning of cultivation (here: t0, inoculation point) until the maximum RFUs and, consequently, the maximum EmGFP concentration are reached (here: t9, after 19 h of cultivation).

### Method validation according to FDA

The validation of the fluorescence-based quantification method was carried out in accordance with the US Department of Health and Human Services Food and Drug Administration, Guidance for Industry “Analytical Procedures and Methods Validation for Drugs and Biologics” (US "FDA" 2015). The goal was to ensure that the assay is reliable, accurate, and reproducible when applied to *E. coli* BL21 pRSET_J23108_EmGFP cells and diluted to OD_600_ 0.0–2.0 cultures expressing EmGFP (data presented in this study) and *E. coli* BL21 without plasmid as a control, using *in-house* EmGFP standard and sodium fluorescein (Na-F*) as a calibration standard. Validation focused on key analytical performance parameters, including linearity of the fluorescence response, limits of detection (LOD) and quantification (LOQ), accuracy of measurement in cell-based matrices, recovery of spiked analytes, and precision in terms of intra- and interday variability. These criteria were evaluated to confirm the suitability of the method for quantitative fluorescence analysis in microbial systems. Cultivation and fluorescence measurements were performed as described in section “Materials and methods,” “Fluorescence measurement.”

#### Standards

Standards and references listed in Table 1 used in this work were commercially purchased (sodium fluorescein [Na-F*], supplier: Carl Roth, GFP supplier: Merck) or prepared *in-house* (EmGFP, see section “*In-house* EmGFP standard preparation”). Prior to use, all standards were handled identically as the samples drawn from cultivation and characterized *in-house* to confirm identity and quality. Fluorescence properties were verified using excitation and emission scans. Protein concentrations were determined via protein absorbance assays. The validated standards were subsequently used as references for both fluorescence-based quantification and SDS-PAGE analysis, enabling consistent calibration and cross-method comparability.

#### Linearity

The linearity of the fluorescence assay was evaluated by measuring RFUs in black 96-well plates of *in-house* EmGFP standard (stock solution 12.6 g/L, diluted to 1:100 126 mg/L; 1:200 63.0 mg/L; 1:267 47.3 mg/L; 1:400 31.5 mg/L; 1:667 18.9 mg/L; 1:1000 12.6 mg/L; 1:1250 10.1 mg/L; 1:2000 6.30 mg/L in PBS, pH 7) and sodium fluorescein (Na-F*, stock solution 2.00 µM equivalent to 0.752 mg/L in 100 mM NaOH, diluted to 1:1.3 1.50 µM; 1:1 1.00 µM; 1:2.7 0.75 µM; 1:4 0.50 µM; 1:8 0.25 µM; 1:20 0.10 µM, pH 13). Further, the fluorescence signal in RFUs of *E. coli* BL21 pRSET_J23108_EmGFP cells (OD_600_ 2.0 of cell suspension equivalent to 16 mg/L EmGFP, diluted to 1:1.3 OD_600_ 1.50; 1:2 OD_600_ 1.00; 1:2.7 OD_600_ 0.75; 1:4 OD_600_ 0.50; 1:6.7 OD_600_ 0.3; 1:10 OD_600_ 0.20; 1:20 OD_600_ 0.10 in PBS, pH 7) was measured. A linear correlation (*R*^2^ 0.99) for RFUs [-] and samples was determined from triplicates, as shown in Supplementary Information, Figs. S8, S9, and S10.

#### LOD and LOQ

The limit of detection (LOD) and limit of quantification (LOQ) were determined based on the standard deviation of the samples at their lowest concentration: *in-house* EmGFP standard dilution 1:2000, *E. coli* BL21 pRSET_J23108_EmGFP dilution 1:20, Na-F* dilution 1:20. LOD and LOQ were calculated using Eq. 1 and Eq. 2.

1 $$\text{LOD }= 3.3 \sigma /S$$

2 $$\text{LOQ }= 10 \sigma /S$$

where σ is the standard deviation and *S* is the slope of the calibration curve.

#### Accuracy and recovery

Accuracy was assessed by spiking known concentrations of sodium fluorescein (Na-F*) in 100 mM NaOH and *in-house* EmGFP standard with *E. coli* BL21 pRSET_J23108_EmGFP in PBS cell suspensions measuring RFUs in black 96-well plates. *E. coli* BL21 pRSET_J23108_EmGFP cell suspensions in PBS dilution 1:2.7–1:20 were spiked with Na-F* (dilution 1:27, 1:4, 1:8, 1:20) in 100 mM NaOH and *in-house* EmGFP standard in PBS (dilution 1:200, 1:267, 1:400, 1:667, 1:1000, 1:1250, 1:2000), respectively.

Recovery (%) from triplicates was calculated to evaluate matrix effects, indicating minimal signal interference from the biological background.

#### Precision

Precision was assessed through intraday (repeatability) and interday (reproducibility) measurements of replicates of Na-F* (dilution 1:27, 1:4, 1:8, 1:20), *in-house* EmGFP standard (dilutions 1:200, 1:400, 1:667, 1:2000), and *E. coli* BL21 pRSET_J23108_EmGFP cultures in PBS at OD_600_ 2.21, 1.48, 0.74. Intraday precision was evaluated by relative standard deviation (RSD) [%], analyzing multiple replicates within the same day, while interday precision was performed over three separate days when stored at 4 °C.

## Results

### Time course of shake flask cultivation

**Fig. 2:**
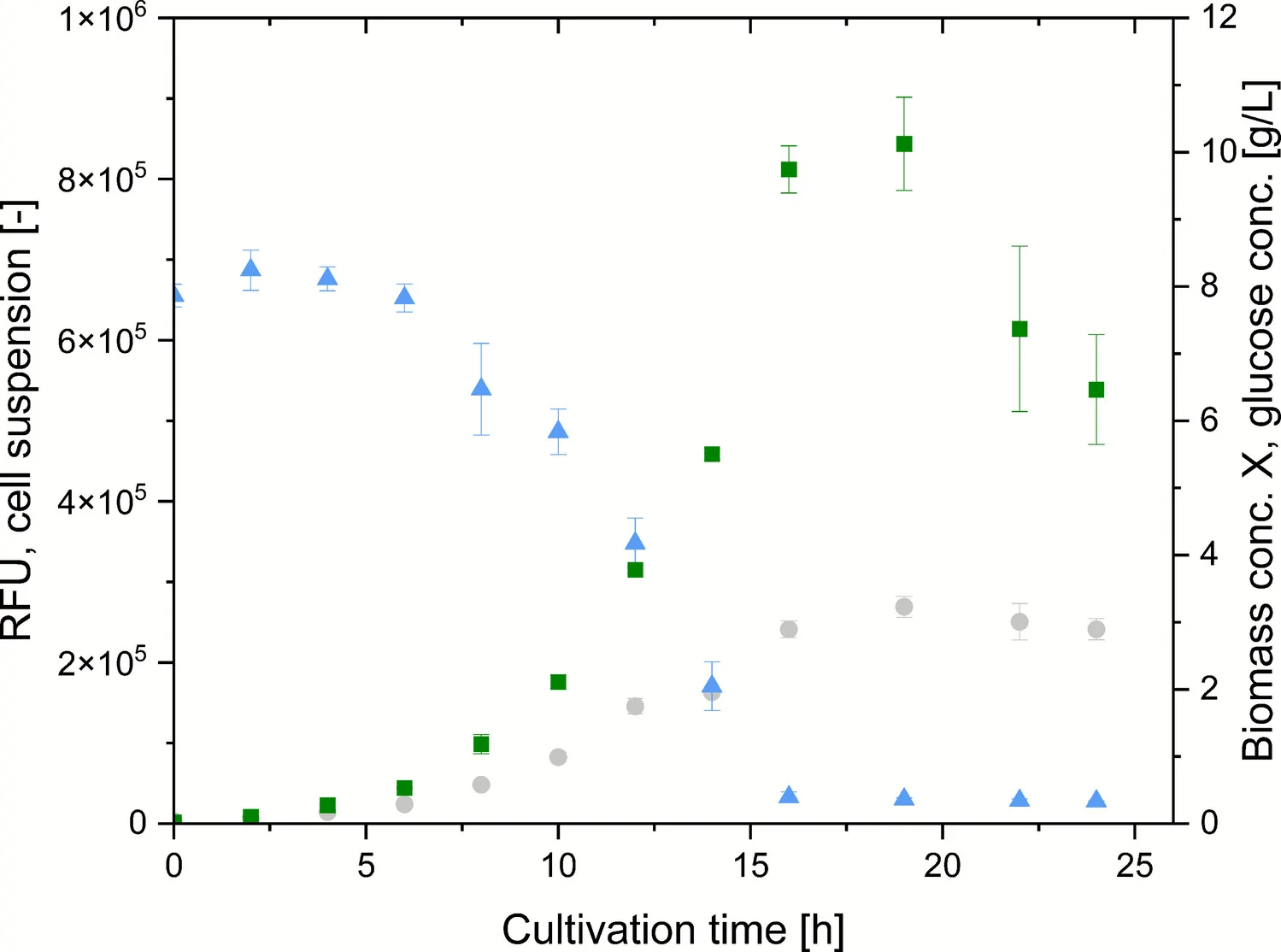
Shake flask cultivation of *E. coli* BL21 pRSET_J23108_EmGFP over 24 h. Time course of biomass concentration X [g/L] (light grey, circles), glucose concentration [g/L] (blue, triangles), and relative fluorescence units of cell suspension (RFU_cellsuspension_) [-] (green, squares)

The shake flask cultivation of *E. coli* BL21 pRSET_J23108_EmGFP at 35 °C in M9 media with an initial glucose concentration of 10 g/L is shown in Fig. 2. Cultivation was monitored over 24 h, and samples were drawn and analyzed every 2 h if not stated otherwise. The substrate was depleted after 16 h (< 0.50 ± 0.05 g/L glucose). Biomass concentration X and RFU_cellsuspension_ peaked at 19 h (3.23 ± 0.16 g/L and [8.44 ± 0.58]*10^5^ [-]). As a control, *E. coli* BL21 without carrying a plasmid was cultivated at 35 °C in M9 media with a starting glucose concentration of 10 g/L (data provided in Supplementary Information, Fig. S3).

### Cell lysis efficiency

To quantify lysis efficiency, colony forming units (CFUs) ranging from 30 to 300 (Breed and Dotterrer 1916) were counted on each plate, accounting for non-lysed functional cells containing EmGFP. Results show that cell lysis had a lysis efficiency of > 99.99% for tested time points t0 (time of inoculation), t5 (after 10 h cultivation time), and t11 (after 24 h cultivation time) (data provided in Supplementary Information, Table S1). Additionally, lysis efficiency was investigated qualitatively with a microscope, showing blurred contours and diffused shapes compared to the control.

### Protein quantification

Two different assays (BCA and Bradford) based on BSA calibration curves (linear fitting *R*^2^ 0.99 provided in Supplementary Information, Figs. S6 and S7) were performed to determine total protein as BSA equivalents, here described as host cell protein (HCP) content of *E. coli* BL21 pRSET_J23108_EmGFP (data provided in Supplementary Information, Fig. S4a). Alike biomass concentration and fluorescence values, HCP peaked after 19 h (as measured according to the Bradford assay), whereas the BCA assay showed maximum HCP after 24 h of cultivation time. The BCA assay yielded a maximum HCP of 2655.4 ± 182.4 mg/L. In contrast, with the Bradford assay, a maximum of 1275.2 ± 290.1 mg/L was measured. As a control, host cell protein of *E. coli* BL21 without plasmid was determined using the BCA and Bradford assays (data provided in Supplementary Information, Fig. S4b).

### Determination of EmGFP concentration via lane intensity from SDS-PAGE

**Fig. 3:**
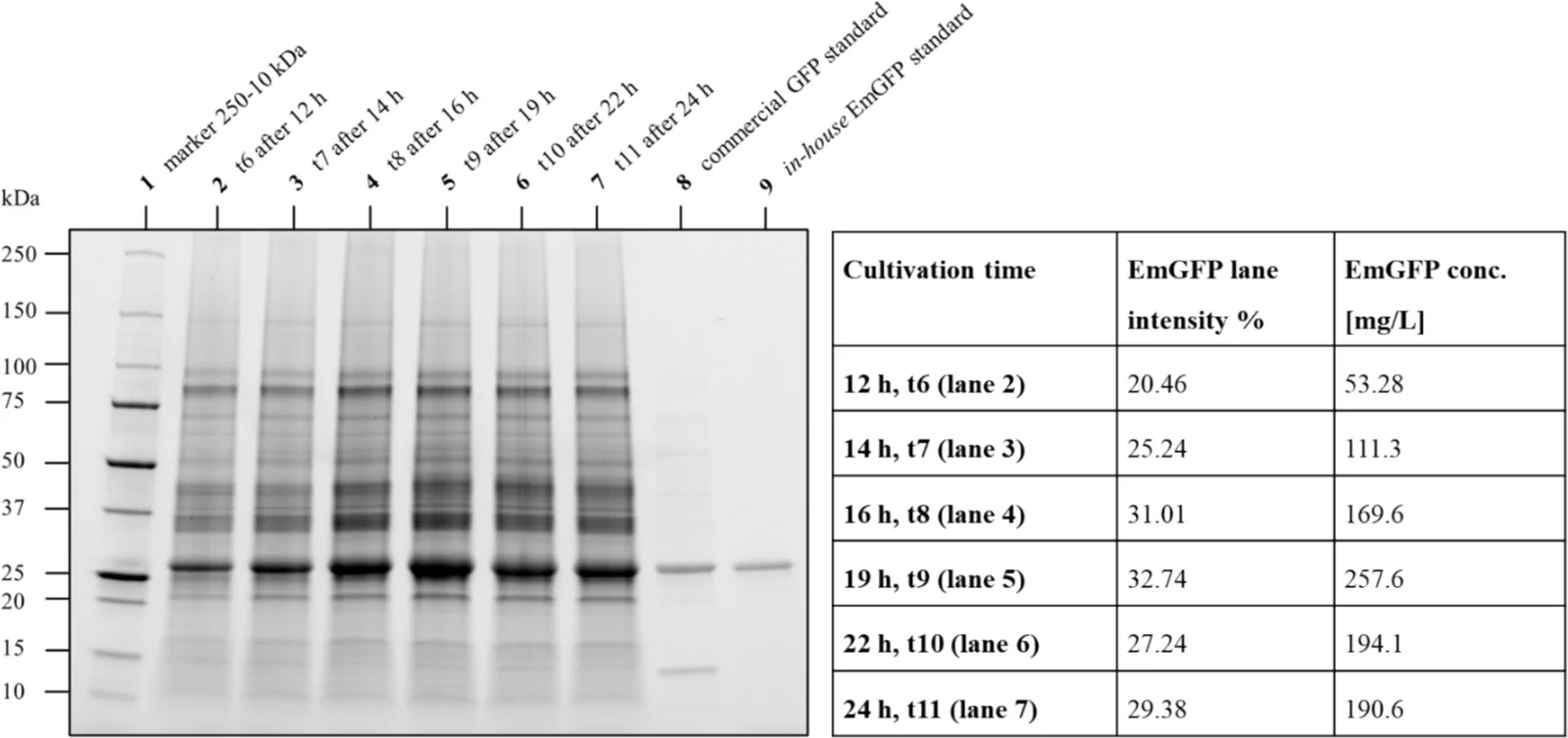
SDS-PAGE gel of culture samples *E. coli* BL21 pRSET_J23108_EmGFP (replicate 1 as an example). Lane 1, molecular weight marker 250–10 kDa; lanes 2–7, time points t6–t11 (t6 after 12 h, t7 after 14 h, t8 after 16 h, t9 after 19 h, t10 after 22 h, t11 after 24 h); lane 8, commercial GFP standard (26.9 kDa); lane 9, *in-house* EmGFP standard (26.9 kDa). The analysis table presents corresponding EmGFP lane intensities (%) and calculated EmGFP concentration in mg/L for time points t6–t11

EmGFP concentration was determined via SDS-PAGE gel visualization. Culture samples of *E. coli* BL21 pRSET_J23108_EmGFP were loaded on the gel and analyzed using software Image Lab (Version 6.1.0 build 7, 2020, Bio-Rad Laboratories, Inc.). An example (*E. coli* BL21 pRSET_J23108_EmGFP replicate 1) is given in Fig. 3 showing an SDS-PAGE gel and calculated EmGFP lane intensities (%) and concentrations [mg/L]. Quantification of lane intensities at the expected molecular weight (approx. 27 kDa), indicative of recombinant EmGFP, was used to estimate EmGFP concentrations in the samples. Lanes 2 to 7 contain cultivation samples of *E. coli* BL21 pRSET_J23108_EmGFP between 12 and 24 h of cultivation (t6–t11). Image Lab pixel density analysis of gel bands at approx. 27 kDa showed that peak EmGFP concentration was reached after 19 h of cultivation (see Fig. 3 (lane 5)). Subsequently, the determination method “lane intensity (%)” is used in Image Lab to relate the selected band (EmGFP) to all bands in the same lane representing protein amount loaded on the lane. For calculations, HCP concentration, loaded sample mass, and buffer ratio are considered. In Fig. 3, an exemplary conversion of EmGFP lane intensity in % to concentration in mg/L is shown (replicate 1). As a result, the maximum EmGFP concentration of 257.6 mg/L was determined at 19 h cultivation time for replicate 1. When determining EmGFP concentration of *E. coli* BL21 pRSET_J23108_EmGFP (*n* = 3), a maximum recombinant EmGFP production of amount host cell protein is given after 19 h resulting in 194.7 ± 44.9 mg/L EmGFP of a total of 1275.2 ± 290.1 mg/L HCP (Bradford assay) content.

###  Relative fluorescent units (RFU) [-] of cell suspension in relation to soluble protein fraction and absolute EmGFP concentration [mg/L]

**Fig. 4:**
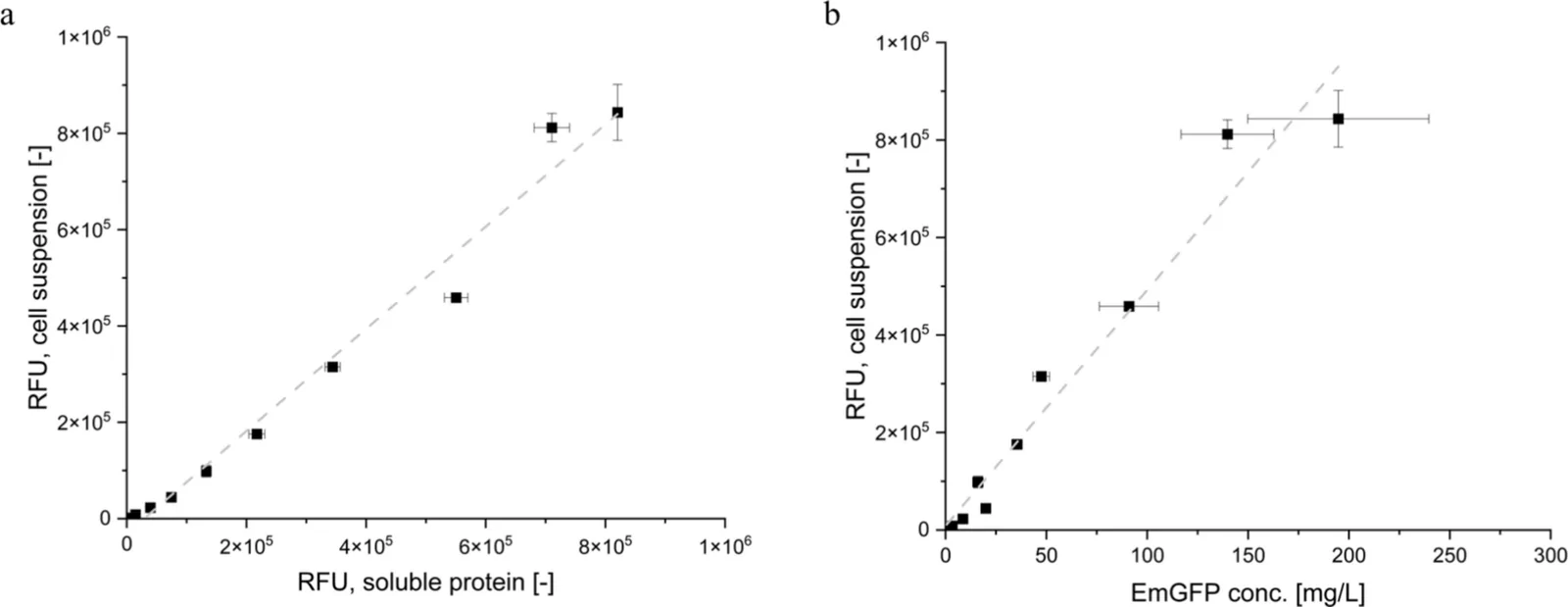
**a** RFU [-] correlation of same time points of cultivation of EmGFP in-cell measurement (cell suspension) and soluble protein fraction (after cell lysis) of *E. coli* BL21 pRSET_J23108_EmGFP (linear fitting, R^2^ 0.98, grey dashed line, y = 1.06125×−30,325.4). **b **RFU of EmGFP in-cell measurement (cell suspension) [-] and EmGFP concentration [mg/L] calculated from lane intensity % of SDS-PAGE correlation of *E. coli *BL21 pRSET_J23108_EmGFP (linear fitting, R^2^ 0.96, grey dashed line, y = 4837.0x+8697.6)

Results of EmGFP in-cell measurement (cell suspension, Fig. 4a *y*-axis, see also Fig. 1 (3b)) compared to soluble EmGFP fraction released after complete cell lysis (99.99%, Fig. 4a *x*-axis, see also Fig. 1 (4b)) reveal similar RFU values for samples for the same time points (e.g., cell suspension (4.59 ± 0.07)*10^5^ RFUs and soluble protein fraction (5.50 ± 0.20)*10^5^ RFUs after 14 h). After 19 h of cultivation, i.e., timepoint t9, maximum RFU was reached for EmGFP measured from cell suspension (8.44 ± 0.58)*10^5^ RFUs and soluble protein fraction (8.20 ± 0.05)*10^5^ RFUs. Although EmGFP detection may be affected when measured inside a cell, it can be concluded that, in this case, the impact was less than 12% and can therefore be neglected. Overall, the confirmed correlation (linear fitting *R*^2^ 0.98) allows direct conversion of RFUs (cell suspension) and later determined EmGFP concentrations, leaving out an additional correlation step with RFUs from soluble protein fraction. EmGFP in-cell measurement (cell suspension) measured in RFU [-] and quantified absolute EmGFP concentration [mg/L] via SDS-PAGE is visualized in Fig. 4b (linear fitting *R*^2^ 0.96). Established correlation allows direct and fast conversion of RFUs from cell suspension into absolute EmGFP concentrations. Maximum EmGFP concentration is reached after 19 h, resulting in 194.7 ± 44.9 mg/L. Vice versa, a maximum of (8.44 ± 0.58)*10^5^ RFUs is measured at the same time point (t9, after 19 h of cultivation), proving robust correlation.

**Fig. 5:**
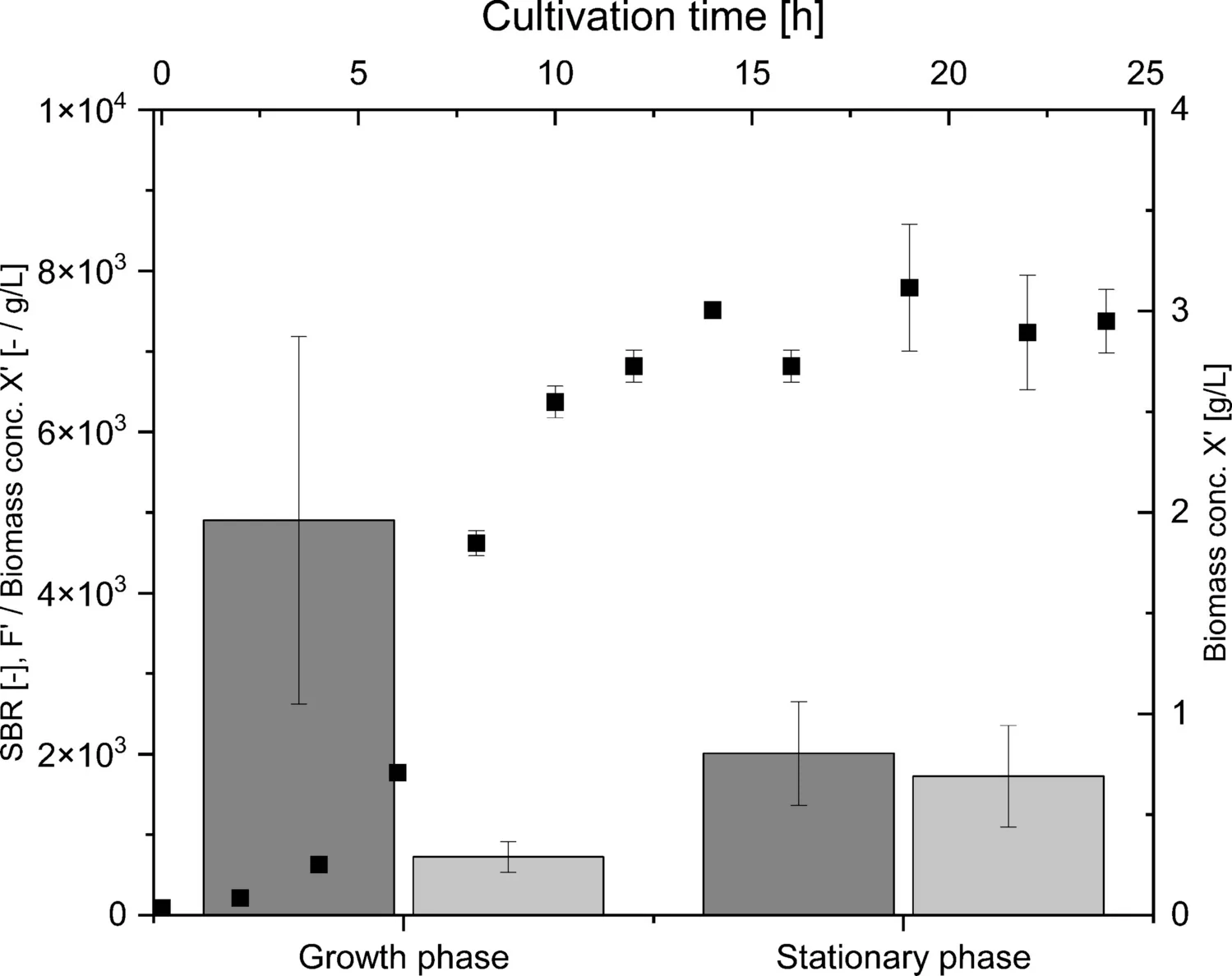
Signal to background (SBR) ratio [-] presented in dark grey bars and autofluorescence of *E. coli* BL21 without a plasmid (F′, cell suspension) per biomass concentration of *E. coli* BL21 without a plasmid (X′) [-/g/L] presented in light grey bars for the growth phase (0 h cultivation time) and the stationary phase (12–24 h cultivation time). Time course of biomass concentration of *E. coli* BL21 without a plasmid (X′) [g/L] (squares) over 24 h

Fluorescence measurements of *E. coli* BL21 cells without a plasmid, i.e., autofluorescence of *E. coli* BL21 cells, are presented in Supplementary Information, Fig. S3, reaching a maximum of (4.64 ± 0.36) *10^3^ RFUs. Over the cultivation time of 24 h, the fluorescence signal of the cell suspension of *E. coli* BL21 without a plasmid and any other potential fluorescence media compounds (here: background, BR) does not exceed 4% at any time point during the cultivation of the recorded fluorescence signal of the cell suspension of *E. coli* BL21 pRSET_J23108_EmGFP (here: signal, S). Figure 5 presents the signal to background (SBR) ratio [-] for the defined growth phase (cultivation time 0–10 h, here: after 10 h, glucose was completely depleted) with an average of (4.90 ± 2.28)*10^3^ RFUs and for the defined stationary phase (cultivation time 12–24 h) with an average of (2.01 ± 0.64)*10^3^ RFUs. Furthermore, the ratio of the autofluorescence of the cell suspension of *E. coli* BL21 cells without a plasmid (F′) to the biomass concentration of *E. coli* BL21 without a plasmid (X′) [-/g/L] is shown with an average of (0.72 ± 0.19)*10^3^ (RFUs/g/L) for the growth phase and (1.73 ± 0.63)*10^3^ (RFUs/g/L) for the stationary phase, as well as the time course of biomass concentration X′ [g/L] over the cultivation time of 24 h, reaching a maximum of 3.12 ± 0.31 g/L after 19 h.

#### Method validation based on FDA requirements

Detailed information and characteristics of standards used for method validation are presented in Table 1 meeting the “Analytical Procedures and Methods Validation for Drugs and Biologics” (US Fda 2015) guideline.

**Table 1:** Characteristic and parameters of standards used in this study (Tsien 1998; Shaner et al. 2005)

Standard	Additional information and supplier	Excitation maxima (nm)	Emission maxima (nm)	Mass	Extinction coefficient ɛ (M^−1^ cm^−1)^	Purity %	Stock conc. and measured RFUs [-]
*In-house* EmGFP standard	Commercial plasmid In-house purification	487	509	26.9 kDa	57,500	78% (from SDS-PAGE)	12.6 ± 0.0 g/L from A_487_ (*n* = 3), 49,043.8 ± 2009.7 for dilution 1:200, 63.0 mg/L
GFP standard	Commercial standard	395/475	509	26.9 kDa	25,000	> 70% (from SDS-PAGE)	1.0 g/L, 40,662.7 ± 894.3 for dilution 1:2, 500 mg/L
Sodium fluorescein (Na-F*)	IUPAC name: 3′,6′-dihydroxyspiro[isobenzofuran-1(3*H*),9′-[9*H*]xanthen]−3-one Commercial standard	485	514	332.31 g/mol	93,000	-	2 μM equivalent to 0.752 mg/L in 100 mM NaOH, 49,102.7 ± 666.9 for dilution 1:2, 0.376 mg/L in 100 mM NaOH

### Linearity, limit of detection (LOD), and limit of quantification (LOQ)

The calibration curves (provided in Supplementary Information, Figs. S8, S9, and S10) showed a linear correlation (*R*^2^ 0.99, *n* = 3) between relative fluorescence units (RFU) [-] and EmGFP concentration (stock solution 12.6 g/L, diluted 1:100, 1:200, 1:267, 1:400, 1:667, 1:1000, 1:1250, 1:2000 in PBS, pH 7), Na-F* (Na-F*, stock solution 2.00 µM equivalent to 0.752 mg/L in 100 mM NaOH, diluted 1:13, 1:1, 1:2.7, 1:4, 1:8, 1:20, pH 13), and *E. coli* BL21 pRSET_J23108_EmGFP cells (OD_600_ 2.0 of cell suspension equivalent to 16 mg/L EmGFP concentration, diluted 1:1.3, 1:2, 1:2.7, 1:4, 1:6.7, 1:10, 1:20 in PBS, pH 7) within the working range.

LOD and LOQ were calculated from established calibration curves (see Table 2). Determined LOD of *E. coli* BL21 pRSET_J23108_EmGFP cells in PBS resulted in OD_600_ 0.03 and a LOQ of OD_600_ 0.08 for tested OD_600_ 0.10. *In-house* EmGFP standard [mg/L] in PBS resulted in an estimated LOD of 0.88 mg/L and a LOQ of 2.66 mg/L for tested concentration 12.6 mg/L EmGFP. Na-F* standard in 100 mM NaOH resulted in an estimated LOD of 0.005 μM and a LOQ of 0.016 μM for tested concentration 0.10 μM.

**Table 2:** Overview of linearity, LOD, and LOQ determined for standards used in this study

	**EmGFP concentration (mg/L)**	***E. coli* BL21 pRSET_J23108_EmGFP OD**_**600**_	Na-F* in 100 mM NaOH
Concentration	12.6	0.10 equivalent to 0.5 mg/L EmGFP	0.1 μM equivalent to 0.0376 mg/L
*R*^2^	0.99	0.99	0.99
LOD	0.88	0.03	0.005
LOQ	2.66	0.08	0.016

### Accuracy and recovery

Four different spiking concentrations of EmGFP standard in PBS (dilution 1:200, 1:267, 1:400, 1:667, 1:1000, 1:1250, 1:2000) were evaluated when mixed with *E. coli* BL21 pRSET_J23108_EmGFP cell suspension 1:2.7–1:20 diluted in PBS. Recoveries of ± 15% (*n* = 3) were achieved, resulting in an overall recovery of <  ± 10%. Results are provided in Supplementary Information, Table S3. Due to the pH sensitivity of the fluorescent measurement and different pH values of tested spiking standards, EmGFP (7.5 ± 0.2) and NaF* (pH 13.0 ± 0.2), data generated of *E. coli* BL21 pRSET_J23108_EmGFP spiked with Na-F* is discussed in section “Discussion” only.

### Precision

Intraday precision was evaluated by analyzing replicates (*n* = 3) within the same day (3 h difference each), resulting in overall RSD below 5%, whereas interday precision, performed over three separate days at a similar time of the day (*n* = 3, ± 1 h), resulted in overall RSD below 10% for tested concentrations. Relative standard deviation (RSD) of intraday (repeatability) and interday (reproducibility) experiments are provided in Supplementary Information, Table S2.

## Discussion

To enable quantitative analysis of recombinant protein expression, a method was developed to correlate relative fluorescence units of cell suspension (RFU_cellsuspension_) [-] with absolute EmGFP concentrations [mg/L] in *E. coli* BL21. Although the initial establishment of this correlation is experimentally demanding, it provides a robust and reproducible foundation for high-throughput fluorescence-based absolute protein quantification assays.

### Cultivation time courses

Cultivation of *E. coli* BL21 pRSET_J23108_EmGFP and *E. coli* BL21 without carrying a plasmid resulted in a steady biomass concentration increase and the complete depletion of the substrate after 16 h and after 10 h, respectively. An increase of the fluorescence signal (RFU) of the cell suspension of *E. coli* BL21 pRSET_J23108_EmGFP compared to the control (*E. coli* BL21 without carrying a plasmid) over time indicates successful EmGFP expression. Maximum RFU_cellsuspension_ of *E. coli* BL21 pRSET_J23108_EmGFP, i.e., maximum EmGFP production, was observed after 19 h (8.44 ± 0.58*10^5^ [-]) followed by declining RFU_cellsuspension_ until the end of cultivation after 24 h. The observed trend is indicative of proteolytic degradation, e.g., recombinant proteins, as glucose is depleted in this phase of cultivation.

### Cell lysis and protein quantification methods

The study by Benov and Al-Ibraheem (2002) underscores the critical role of cell lysis methods and their efficiency in *E. coli* when targeting recombinant protein purification. In addition, the study provides a comparative evaluation of various total protein quantification techniques and discusses their different performances (Wall and Gehrke 1975; Biemann and Martin 1987; Benov and Al-Ibraheem 2002; Mariotti et al. 2008; De Mey et al. 2008). Cell lysis and efficiency were assessed as incomplete lysis or incompatible methods with subsequent assays or downstream analyses, e.g., absorbance-based protein quantification, protein purification, or SDS-PAGE may lead to underestimation of HCP concentration and therefore influence quantification of the protein of interest. Additionally, cultivation sample storage conditions were investigated. Here, supplementation with EDTA-free protease inhibitor reagent and 4 °C were selected as the procedure of choice, preventing HCP degradation. Commonly used quantification methods such as the bicinchoninic acid (BCA) assay, Bradford assay, Lowry assay, or absorbance measurement at 280 nm rely on different detection principles. While the BCA assay responds to peptide bonds as well as aromatic residues, with absorbance measured at 562 nm, the Bradford assay is based on dye binding and detects absorbance at 595 nm, primarily reflecting the presence of cationic and non-polar side chains in proteins. Consequently, variations in the resulting protein concentrations are not unexpected but rather imperative to the differing assay compositions. Although GFP (represented here by EmGFP) exhibits low absorbance in the 550–650 nm range, it has its maximum absorbance at approximately 487 nm (for EmGFP), and therefore, interference should not be neglected (Yakhnin et al. 1998). Based on values from the literature describing 40–60% HCP (Matassa et al. 2016) of the biomass concentration (here 3.23 ± 0.16 g/L), the BCA assay determination method was excluded from further investigation, as the values appear to be outside the expected range (> 60% HCP of biomass concentration, data provided in Supplementary Information, Fig. S4). Protein quantification analysis was performed using the results obtained from the Bradford assay only. In the subsequent step, EmGFP production was analyzed using SDS-PAGE imaging, which detected bands at the expected molecular weight of approx. 27 kDa, confirming the presence of EmGFP. Depending on the expression system used, it has been reported that the recombinant protein expression can reach up to 50% of the HCP (Miroux and Walker 1996; Baneyx 1999; Matassa et al. 2016). In the present study, a maximum of approx. 15% of recombinant EmGFP concentration per HCP was reached after 19 h (194.7 ± 44.9 mg/L EmGFP per 1275.2 ± 290.1 mg/L HCP, data provided in Supplementary Information, Fig. S2). To identify potential housekeeping proteins migrating at the same molecular weight as the target protein, *E. coli* BL21 without a plasmid was included as a negative control (data provided upon request). Although faint bands were detected around approx. 27 kDa, their intensity consistently remained below 5% of the total protein signal over the entire cultivation time. These signals are likely attributable to constitutively expressed housekeeping genes such as small heat shock proteins, transcription factors, or metabolic enzymes typically found in the 20–30 kDa range (Kappé et al. 2002; Grainger et al. 2007; Zhou et al. 2011). Due to their low abundance and consistent expression, these bands were not considered in the quantification of EmGFP concentration, where they are likely overlapped by the significantly stronger target protein band.

### Fluorescent proteins and factors influencing detection and fluorophore stability

It is important to note that ensuring reproducibility across fluorescence analysis is of great importance. While the presented approach is designed to be conducted with common laboratory methods and applicable to other cultivation settings (e.g., cell types, here *E. coli*, different cultivation vessels, cultivation and expression temperatures, or plasmid and GFP variants), metabolic burden and differences in expression (e.g., altered cell shape or expression rate) may influence EmGFP quantification results and should be taken into account. In addition, the state of the fluorescent protein (e.g., localized as inclusion bodies, secreted into the medium, or retained intracellularly) and proper, consistent, and stable folding of the fluorescent protein under defined conditions is crucial, as it directly influences signal intensity (Zimmer 2002; Wachter 2006). Furthermore, the presence of oxygen is crucial for fluorescent proteins to function as real-time reporters (Drepper et al. 2010). Additional fluorophore-specific effects such as quenching, affected by protein concentration, sample dilution, or buffer composition (e.g., the presence of Cu^2^⁺ ions) can further influence fluorescence signal recording (Richmond et al. 2000; Jung, Kie-Chul et al. 2005). By maintaining consistent experimental conditions, including media composition, cultivation vessel and lid geometry, temperature of preparation, cultivation, analysis, pH value, constant oxygen availability, expression strain, and sampling procedure, potential influences on the assay were minimized. These measures contribute to the robustness and reproducibility of the assay and support its applicability across comparable conditions. Furthermore, fluorescence intensity of many fluorophores is pH dependent. Consequently, fluorescent measurement at pH conditions suiting both references and samples might be a hurdle to overcome (Jung, Kie-Chul et al. 2005; Ishii et al. 2007). *In-house* EmGFP standard [mg/L] and *E. coli* BL21 pRSET_J23108_EmGFP (OD_600_) were tested in PBS at pH 7.5, 9.5, and 13.0 each with a deviation of ± 0.2 using 1.0 M NaOH titration, while Na-F* in 100 mM NaOH was tested at pH 13.0, 9.5, 7.5, and 5.0 each with a deviation of ± 0.2 using 1.0 M HCl titration. As reported by Ishii et al. (2007), the fluorescence response of all tested fluorophores was strongly influenced by pH, confirming their pH sensitivity. Whereas *in-house* EmGFP standard and *E. coli* BL21 pRSET_J23108_EmGFP showed stable RFU at pH 7.5 and 9.5 each with a deviation of ± 0.2, tested pH 13.0 ± 0.2 resulted in reduced RFU values. In comparison, Na-F* showed stable RFU at pH 9.5 and 13.0 each with a deviation of ± 0.2, but decreased fluorescence intensity at pH 7.5 and 5.0 each with a deviation of ± 0.2 (data provided by request, see also section “Accuracy and Recovery” in “Results”).

### Absolute EmGFP quantification via relative fluorescent units (RFU)

Importantly, only data points within the defined correlation range can be converted into absolute quantitative values here, up to a maximum of (8.44 ± 0.58) *10^5^ [-], *R*^2^ = 0.96. Extrapolation beyond this range should be strictly avoided; instead, the calibration curve should be systematically extended to cover the desired measurement interval. In this study, EmGFP was expressed as the target fluorescent protein, and due to its spectral similarity (fluorescence spectrum scan provided in Supplementary Information, Fig. S11), sodium fluorescein (Na-F*) was selected as an additional chemical standard. Parameters and conditions (such as plate type, filling volume, mixing of the sample, gain settings, or Z-position) were consistent over all measurements. Moreover, it was essential to verify whether culture media, dilution solutions, or cells without carrying a plasmid, i.e., no EmGFP expression, result in neglectable relative fluorescence units (RFUs). Background signals and photobleaching occurring naturally can impact the accuracy of the correlation (White 1999; Zimmer 2002; Wachter 2006) and were evaluated for each experimental setup as described in section “Fluorescence measurement” and shown in Fig. 5. In this study, samples were stored at 4 °C in PBS covered from light.

When evaluating the fluorescence signal from microorganisms, it is essential to take autofluorescence into account. Cells appear to exhibit natural fluorescence, potentially disturbing measurements or delivering misleading results (Billinton and Knight 2001). The signal to background (SBR) ratio was investigated. A higher SBR ratio in the growth phase is likely due to increased metabolic activity of both *E. coli* BL21 pRSET_J23108_EmGFP and without a plasmid. Cells maintain their structural integrity in the growth phase (no significant release of intracellular fluorescent compounds), and *E. coli* BL21 pRSET_J23108_EmGFP produces its target protein EmGFP in high amounts. In the stationary phase, substrate is depleted; *E. coli* BL21 pRSET_J23108_EmGFP cells subsequently stop producing EmGFP, *E. coli* BL21 cells and proteins partly lyse, and intrinsic compounds are released to the media. So-called “green-emitters” that have to be considered are e.g. flavins, NAD(P)H, or collagen (Aubin 1979; Benson et al. 1979; Billinton and Knight 2001). Due to cell debris, the accumulation of cell molecules potentially being fluorescence emitters, and reduced light scattering, a slight steady increase of fluorescence signals even after substrate depletion can appear. Autofluorescence of *E. coli BL21* without a plasmid (F′) to biomass concentration (X′) ratio shows similar ratios for the growth phase and the stationary phase, indicating a constant fluorescence signal emission per biomass concentration produced. The slight increase in the stationary phase is most likely due to the aspects mentioned above. Overall results show (< 4% F′ of S over the entire cultivation time and similar RFU values of the same time points of cultivation of EmGFP in-cell measurement and soluble protein fraction, data provided in Fig. 5a, Fig. 5, and in Supplementary Information, Fig. S3) that the autofluorescence of *E. coli* BL21 cells can be neglected in the present study, but it is essential to consider these factors when establishing correlations between fluorescence intensity and absolute protein concentration. It is recommended to establish a correlation between RFUs of cell suspension and absolute FP concentration once for a defined experimental setup. Once validated, the correlation can be reliably applied in subsequent experiments as a high-throughput method for absolute protein quantification for tested cultivation conditions and setup.

### FDA method validation requirements

The presented method was successfully validated according to “Analytical Procedures and Methods Validation for Drugs and Biologics” (US "FDA" 2015). The linearity (*R*^2^ 0.99, *n* = 3) confirms that the detector response is proportional to analyte concentration under the given conditions and supports the use of RFU for quantitative analysis within this range. LOD (*E. coli* BL21 pRSET_J23108_EmGFP cells in PBS: OD_600_ 0.03, *in-house* EmGFP standard 0.88 mg/L in PBS, and Na-F* standard 0.005 μM in 100 mM NaOH) and LOQ (*E. coli* BL21 pRSET_J23108_EmGFP cells in PBS: OD_600_ 0.08, *in-house* EmGFP standard 2.66 mg/L in PBS, and Na-F* standard 0.016 μM in 100 mM NaOH) were determined for tested concentrations of *E. coli* BL21 pRSET_J23108_EmGFP cells in PBS OD_600_ 0.1, *in-house* EmGFP standard 12.6 mg/L in PBS, and Na-F* standard 0.10 μM in 100 mM NaOH.

This approach ensures sufficient sensitivity to detect low-level fluorescence in conducted assays. Furthermore, the determined overall recovery of <  ± 10%, the intraday (repeatability) of < 5%, and the interday (reproducibility) of < 10% precision met the requirements of the guidelines (recovery ± 15%, intraday > 10%, interday < 20%) (US "FDA" 2015, 2018).

## Conclusion

Within this work, a robust and reproducible approach for the absolute quantification of fluorescent protein was presented, using the platform example of Emerald Green Fluorescent Protein (EmGFP) expressed in *E. coli* BL21. By combining fluorescence measurements, host cell protein analysis, and standard-based calibration, relative fluorescence units (RFUs) of in-cell measurements (cell suspension) can be reliably converted into absolute EmGFP concentrations (mg/L). The method was optimized and validated according to FDA guidelines “Analytical Procedures and Methods Validation for Drugs and Biologics,” demonstrating high linearity, precision, and accuracy. Moreover, commercial standards such as fluorescein and GFP, as well as an *in-house* EmGFP standard, were used for comparability. Its advantage lies in the integration of a microtiter-based format with commonly available laboratory equipment and techniques, enabling efficient quantification without the need for specialized instrumentation. This standardized workflow allows straightforward comparison when applied across cultivation scales and facilitates rapid assessment of productivity under varying conditions. Beyond its application in laboratory-scale research, the method also holds strong potential for precision fermentation, where quantitative monitoring of recombinant protein expression and product yields is crucial for process design and optimization. Overall, this method offers a versatile, scalable, and accessible tool for quantitative fluorescence-based protein analysis in protein production research.

## Supplementary Information

Below is the link to the electronic supplementary material.

ESM 1 (DOCX 2.12 MB)

ESM 2 (JPG 196 KB)

## Data Availability

No datasets were generated or analysed during the current study.
