# Supplementary material for: Making GFP count: a validated framework for absolute protein quantification in precision fermentation
Source: Appl Microbiol Biotechnol. 2026 Feb 4;110:56. doi: 10.1007/s00253-026-13734-z (PMC12876113; doi:10.1007/s00253-026-13734-z)
Supplement: Supplementary file 1 [file 253_2026_13734_MOESM1_ESM.docx]

Supplementary Information

**Making GFP Count: A Validated Framework for Absolute Protein Quantification in Precision Fermentation**

Christina Peternell, Philipp Noll, Annette Brümmer-Rolf and Marius Henkel*

*Corresponding author: Marius Henkel

Address: Gregor-Mendel-St. 4, 85354 Freising, Germany

Phone: +49 (8161) 71 - 5130

Email: [marius.henkel@tum.de](mailto:marius.henkel@tum.de)

Plasmid map of expression system used in this study

**Fig. S1** Plasmid map of pRSET_J23108_EmGFP (3321 bp) transformed into *E. coli* BL21 created with *Benchling.com*

Recombinant EmGFP concentration compared to amount of host cell protein

**Fig. S2** Recombinant EmGFP concentration (shaded bar) determined from SDS–PAGE band intensities % from host cell protein (HCP, white bar) determined with Bradford assay in mg/L and biomass concentration X (light grey, circles) in g/L of *E. coli* BL21 pRSET_J23108_EmGFP.

Time course of shake flask cultivation of *E. coli* BL21 (without plasmid) at 35 °C over 24 h

**Fig. S3** Time course of biomass concentration X’- (light grey, circles) and glucose concentration (blue, triangles) [g/L] and relative fluorescence units of cell suspension i.e. autofluorescence (RFU_cellsuspension_) [-] (green, squares) of shake flask cultivation *E. coli* BL21 (without plasmid).

Protein quantification as host cell protein (HCP) with Bicinchoninic acid (BCA) assay and Bradford assay of *E. coli* BL21 pRSET_J23108_EmGFP and *E. coli* BL21 (without plasmid)

**Fig. S4 a** Host cell protein (HCP) [mg/L] of *E. coli* BL21 pRSET_J23108_EmGFP. Bicinchoninic acid (BCA) assay (orange, triangles) and Bradford assay (squares, blue) are illustrated for comparison related to biomass concentration X (light grey, circles) [g/L] **b** Host cell protein (HCP) [mg/L] of *E. coli* BL21 (without plasmid). Bicinchoninic acid (BCA) assay (orange, triangles) and Bradford assay (squares, blue) are illustrated for comparison related to biomass concentration X’ (light grey, circles) [g/L].

Cell lysis efficiency

**Table S1 Results of colony forming units (CFU) based on initial cell concentration before cell lysis for *E. coli* BL21 pRSET_J23108_EmGFP time points t0 (time of inoculation), t5 (after 10 h cultivation time) and t11 (after 24 h cultivation time)**

| **Average OD_600_ [-] of *E. coli* BL21 pRSET_J23108_EmGFP (cultivation timepoint [h])** | **Average [CFU/mL] based on OD_600_**^[1]^ | **Average [CFU/mL] after cell lysis** |
| --- | --- | --- |
| **0.12 ± 0.01 (0)** | 1.23E+07 ± 0.09E+07 | <3.00E+02 ± 0.0E+02 |
| **2.97 ± 0.19 (10)** | 2.97E+08 ± 0.19E+08 | <3.00E+02 ± 0.0E+02 |
| **8.67 ± 0.38 (24)** | 8.67E+08 ± 0.47E+08 | 8.24E+02 ± 0.20E+02 |

Glucose calibration curve for glucose assay

**Fig. S5** Calibration curve (linear fitting R² 0.99, grey dashed line, y= 0.00001x+0.0473) of glucose standard solution 0.0-1000 mg/L and corresponding absorbance values measured at 510 nm (n=3).

BSA calibration curve for Bradford assay

**Fig. S6** BSA calibration curve (linear fitting R² 0.99, grey dashed line, y= 0.0043x+0.6724) of BSA standard solution 0.0-100.0 mg/L and corresponding absorbance values measured at 595 nm (n=3).

BSA calibration curve for Bicinchoninic acid (BCA) assay

**Fig. S7** BSA calibration curve (linear fitting R² 0.99, grey dashed line, y= 0.0011x+0.0811) of BSA standard solution 0.0-1000 mg/L and corresponding absorbance values measured at 562 nm (n=3).

Method validation, linearity of *E. coli* BL21 pRSET_J23108_EmGFP cells

**Fig. S8** Calibration curve (linear fitting R² 0.99, grey dashed line, y= 28983.4x+5.3767) of *E. coli* BL21 pRSET_J23108_EmGFP cells OD_600_ 0.0-2.0 and corresponding RFU [-] (n=3).

Method validation, linearity of *in-house* EmGFP [mg/L] standard

**Fig. S9** Calibration curve (liner fitting R² 0.99, grey dashed line, y= 865252.0x+5.6605) of *in-house* EmGFP 0.0-63.0 mg/L and corresponding RFU [-] (n=3).

Method validation, linearity of sodium fluorescein (Na-F*) [μM] in 100 mM

**Fig. S10** Calibration curve (liner fitting R² 0.99, grey dashed line, y= 48665.6x+8.7543) of sodium fluorescein (Na-F*) 0.0-1.5 μM in 100 mM NaOH and corresponding RFU [-] (n=3).

Precision: intra- and interday analysis

**Table S2 Overview of intraday and interday assay showing standard concentrations, corresponding RFU [-], and relative standard deviation (RSD, %)**

|  |  | **Intraday** | | **Interday** | |
| --- | --- | --- | --- | --- | --- |
| **Standard** | **Concentration** | **RFU [-]** | **RSD%** | **RFU [-]** | **RSD%** |
| **Sodium fluorescein (Na-F*) [μM] in 100 mM NaOH** | **1.0** | 50597.9 ± 1027.3 | 2.03 | 49948.7 ± 697.7 | 1.40 |
|  | **0.75** | 37561.4 ± 786.9 | 2.09 | 37300.3 ± 351.1 | 0.94 |
|  | **0.5** | 27079.8 ± 779.6 | 2.88 | 26917.6 ± 668.3 | 2.48 |
|  | **0.25** | 13612.2 ± 560.4 | 4.12 | 13523.1 ± 527.0 | 3.90 |
|  | **0.0** | 10.6 ± 0.5 | 4.71 | 10.6 ± 0.5 | 4.71 |
| **EmGFP [mg/L]** | **63.0** | 51561.2 ± 1576.7 | 3.06 | 51849.1 ± 1851.6 | 3.57 |
|  | **31.5** | 25437.0 ± 1141.0 | 4.49 | 25682.0 ± 1343.3 | 5.23 |
|  | **10.1** | 5986.8 ± 417.6 | 6.98 | 5880.0 ± 403.9 | 6.87 |
|  | **6.30** | 2807.7 ± 461.5 | 16.4 | 2461.8 ± 134.0 | 5.44 |
| ***E coli.* BL21 pRSET_J23108_EmGFP OD_600_** | **2.21** | 24100.6 ± 815.9 | 3.39 | 23222.4 ± 957.1 | 4.12 |
|  | **1.48** | 41821.8 ± 1875.9 | 4.49 | 39989.6 ± 1030.6 | 2.58 |
|  | **0.74** | 12322.0 ± 304.5 | 2.47 | 11586.9 ± 564.2 | 4.87 |
|  | **0.0** | 5.1 ± 0.3 | 6.15 | 5.2 ± 0.4 | 7.96 |

Recovery

**Table S3 Overview of spiked sample assay. Recovery (%) of four *in-house* EmGFP standard concentration [mg/L] in *E. coli* BL21 pRSET_J23108_EmGFP cell suspension OD_600_ 0.0-0.75**

|  | **Recovery (%) of *in-house* EmGFP** | | | |
| --- | --- | --- | --- | --- |
| ***E. coli* BL21 pRSET_J23108_EmGFP OD_600_** | **63.0 mg/L** | **47.3 mg/L** | **31.5 mg/L** | **18.9 mg/L** |
| **0.75** | 78.1 | 73.1 | 86.1 | 91.3 |
| **0.50** | 85.5 | 88.5 | 93.8 | 106.5 |
| **0.30** | 85.9 | 90.8 | 88.0 | 91.4 |
| **0.25** | 87.2 | 95.3 | 95.0 | 95.9 |
| **0.16** | 89.7 | 87.9 | 95.6 | 94.0 |
| **0.10** | 90.7 | 83.9 | 94.1 | 91.3 |
| **0.0** | 91.2 | 92.8 | 97.2 | 95.7 |

Fluorescence scan of standards used in this study:

**Fig. S11** Emission scan wavelength 350-600 nm, excitation wavelength 487 nm (dotted line) and excitation scan wavelength 350-600 nm, emission wavelength 540 nm (solid line) of standards *in-house* EmGFP (**a**, 0.063 mg/L), commercial GFP standard (**b,** 1.0 g/L) and Na-F* (**c**, 0.5 μM in 100 mM NaOH) over RFU [-]

Structural illustration of standards used in this study

**Fig. S12** **a** 3D model of EmGFP with 6xhis-tag (based on amino acid sequence used in this work) created with Alpha Fold 2 Protein Structure Database (AFDB) (Varadi et al. 2024) & PyMOL Molecular Graphics System, Version 3.1, LLC **b** chemical structure of sodium fluorescein (Na-F*) a fluorescent tracer created with ChemSketch, version 2024, Advanced Chemistry Development, Inc. (ACD/Labs), Toronto, ON, Canada

*In-house* EmGFP standards purification

**Fig. S13** **a** Protein separation and affinity based (6x his-tag linked to the EmGFP) purification with ÄKTA pure 25 and HisTrap column presenting the following steps: sample application (phosphate buffer), washing step (phosphate buffer supplemented with 5 mM imidazole), elution step 1 (phosphate buffer supplemented with 0.4 M imidazole), elution step 2 (phosphate buffer supplemented with 0.5 M imidazole). UV 280nm (grey line) and UV 487 nm (green line) [mAU] is plotted over volume [mL]. Additionally, the gradient in [%] (blue line) is presented over volume [mL]. **b** Absorbance UV 280nm (grey line) and UV 487 nm (green line) [mAU] is plotted over volume [mL] on a desalting column (Superdex200 10/300 GL, Marlborough, MA, USA) for buffer exchange to phosphate buffer without imidazole. Additionally, conductivity [mS/cm] is illustrated over volume [mL]. Fraction 15.9 – 18.2 mL from the elution phase where collected as “EmGFP standard” based on absorbance UV 280nm and UV 487 nm [mAU].

1. For *E. coli* BL21 pRSET_J23108_EmGFP OD_600_ of 1.0 equals 1.0*10^8^ cells, equaling CFU/mL (data provided upon request)
