# Supplementary figures and images for: Making GFP count: a validated framework for absolute protein quantification in precision fermentation
Source: Appl Microbiol Biotechnol. 2026 Feb 4;110(1):56. doi: 10.1007/s00253-026-13734-z (PMC12876113; doi:10.1007/s00253-026-13734-z)

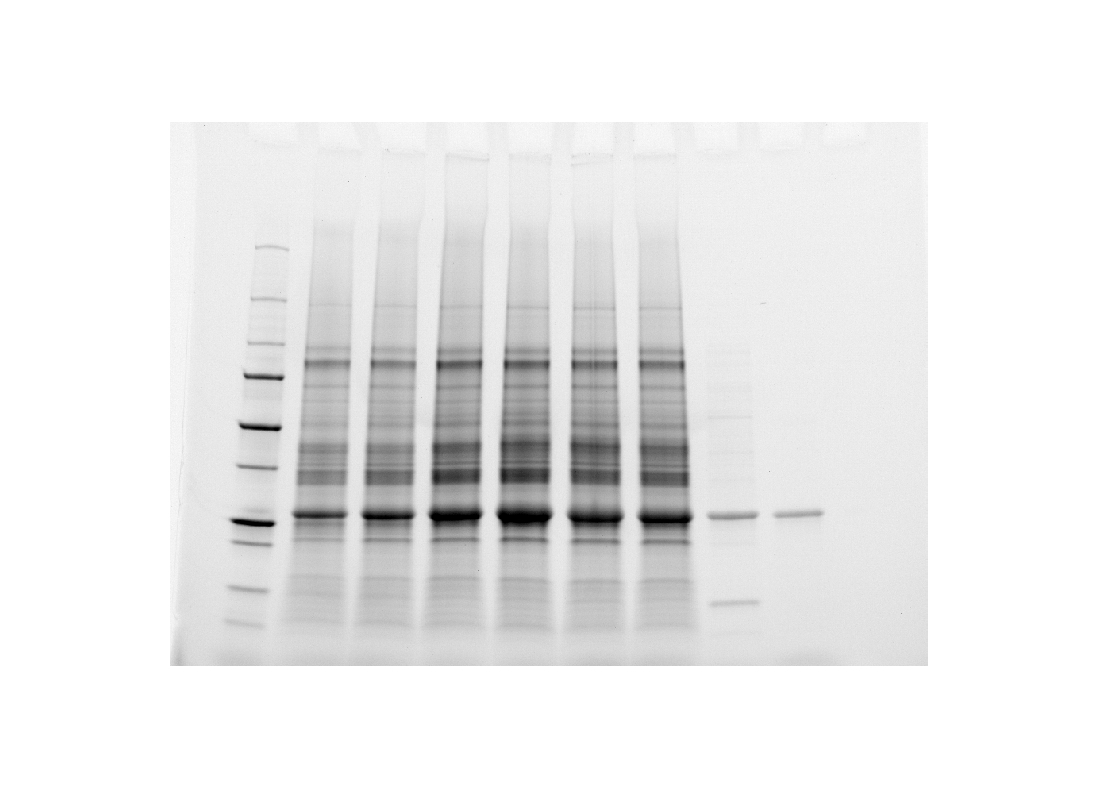

Supplement: Supplementary file 2 — (JPG 196 KB) [file 253_2026_13734_MOESM2_ESM.jpg]
